# Mental Health and Psychosocial Problems among Children and Adolescents in Jordan: A Scoping Review

**DOI:** 10.3390/children10071165

**Published:** 2023-07-04

**Authors:** Rana AlHamawi, Yousef Khader, Sara Abu Khudair, Eizaburo Tanaka, Mohannad Al Nsour

**Affiliations:** 1 Eastern Mediterranean Public Health Network (EMPHNET), Amman 11196, Jordan; 2 Department of Public Health, Jordan University of Science and Technology, Irbid 22110, Jordan; 3 Japan International Cooperation Agency, Amman 11190, Jordan

**Keywords:** mental health, mental health disorders, children and adolescents, psychiatry

## Abstract

**Introduction**: In Jordan, mental health morbidity among children and adolescents is on the rise. Several studies in Jordan have assessed mental health issues and their associated factors among adolescents; however, there remains a lack of a collation of data regarding such issues. **Objectives**: To review the prevalence rates of mental health problems among children and adolescents in Jordan to understand the evidence base concerning psychiatric morbidity. **Methods**: The PubMed database, Cochrane Library, Virtual Health Library (VHL) Lilac, and APA PsycArticles were searched for literature published between January 2010 and May 2023. Studies were included if they were conducted on children and adolescents (≤19 years), were observational studies that reported prevalence data regarding psychosocial problems, and were studies conducted in Jordan. **Results**: The search yielded 211 records, of which 33 studies were assessed for eligibility and 28 met the inclusion criteria. The sample age ranged from 6–19 years. The prevalence rates ranged from 7.1% to 73.8% for depression, 16.3% to 46.8% for anxiety, 13.0–40.6% for ADHD, 11.7–55.2% for overall emotional and behavioral difficulties, 16.2–65.1% for PTSD, and 12–40.4% for eating disorders. **Conclusions**: The findings highlight the magnitude of mental health problems among children and adolescents and the heterogeneity of the results. Further studies are needed to investigate the prevalence of eating disorders among refugees, as well as sleeping disorders and substance use disorders among all adolescents.

## 1. Introduction

Worldwide, the prevalence of mental health problems among adolescents aged 10–19 is high and continues to increase, with a recent review reporting an increase in the age-standardized rate of disability-adjusted life years (DALYs) for mental disorders in children and adolescents from 803.8 per 100,000 to 833.2 per 100,000 from 1990 to 2019 [1]. In 2019, it was estimated that one in seven adolescents experienced a mental health disorder, with anxiety and depressive disorders accounting for 40% of mental disorders, followed by conduct disorders (20.1%) and attention deficit hyperactivity disorder (19.5%) [2]. Globally, the burden of depressive disorders is high among adolescents aged 10–24 [3]. The recent COVID-19 pandemic has further negatively impacted the emotional well-being of adolescents due to its inherent characteristics, such as prolonged school closure, strict social isolation from peers, teachers, extended family, and community networks, and the pandemic itself [4].

In Jordan, mental health morbidity among children and adolescents is on the rise, especially among refugee children. Jordan hosts the second-highest share of refugees per capita in the world, where 48% of refugees are children (UNICEF, 2019) [5]. Refugees are vulnerable to mental health disorders since they are subjected to numerous risks and stressful events. Adolescents, making up 21% of the total Jordanian population, are vulnerable to discrimination, stigma, social exclusion, educational difficulties, risk-taking behaviors, physical ill-health, and human rights violations, which in turn worsens their mental well-being [6].

Many studies [7,8,9,10] in Jordan have assessed mental health issues and their associated factors among adolescents; however, there remains a lack of a collation of data regarding such issues. Therefore, this study aimed to review the prevalence rates of mental health and psychosocial problems among adolescents in Jordan to better understand the evidence base concerning adolescent psychiatric morbidity. The main research question was “What is the available evidence regarding the prevalence of mental health issues among children and adolescents in Jordan?” The objectives are to identify the prevalence and type of morbidity, as well as the associated factors, identify research priorities and gaps, and the problems in primary research that should be rectified in future studies. A scoping review was chosen for this study because this type of review explores the breadth or extent of the literature, maps and summarizes the evidence, and informs future research [11]. Unlike systematic reviews, scoping reviews are not meant to inform practice or policy; instead, they are used to inform future research [11].

## 2. Methods

### 2.1. Literature Search

A preliminary search was conducted using the PubMed database to familiarize ourselves with the literature, refine the aims of the research, and identify relevant keywords and medical subject headings (MeSH terms). The identified keywords were used during the main search (Box 1). The PubMed database, Cochrane Library, and Virtual Health Library (VHL) Lilac were searched on 19 May 2023 for literature published between January 2010 and 2023. In addition, APA PsycAricles was searched for relevant literature. This time period (2010–2023) was chosen since several traumatic events took place in Jordan. For example, in 2011, Syrian refugees chose asylum in Jordan due to the civil war that broke out in March 2011, in addition to the COVID-19 pandemic that started in 2019. The search was not limited by the type of publication or language used. The results were imported into Rayyan where screening of the results was initiated. The Rayyan tool is a web and mobile application to facilitate the screening of articles for scoping reviews [12]. The retained articles were hand searched for relevant literature. The PRISMA-ScR guidelines were followed when preparing the articles.

Box 1 **Search terms used to search the PubMed database from 2010 to May 2023.**

1. “Mental Health” [Mesh] or “Mental illness” [tw] or “Mental Disorders” [Mesh] or anxiety[tw] or “Depression” [Mesh] or “Depressive Disorder” [Mesh] or “Psychosocial problems” [tw] or “psychiatric disorders” [tw] or "Problem Behavior” [Mesh] or “behavioral problem” [tw] or “Conduct Disorder” [Mesh] or “emotional problems” [tw] or “Attention Deficit Disorder with Hyperactivity” [Mesh] or “Adverse Childhood Experiences” [Mesh] or “Adverse childhood experiences” [tw] or fear [tw] or attention-deficit [tw] or “attention deficit” [tw] or “Obsessive-Compulsive Disorder” [Mesh] or “Stress Disorders, Post-Traumatic” [Mesh] or “Intellectual Disability” [Mesh] or “Autistic Disorder” [Mesh] or “Autism Spectrum Disorder” [Mesh]

               AND

2. “Adolescent” [Mesh] or “Child” [Mesh] or child [tw]

               AND

3. “Jordan” [Mesh]

### 2.2. Eligibility Criteria

**Figure 1:**
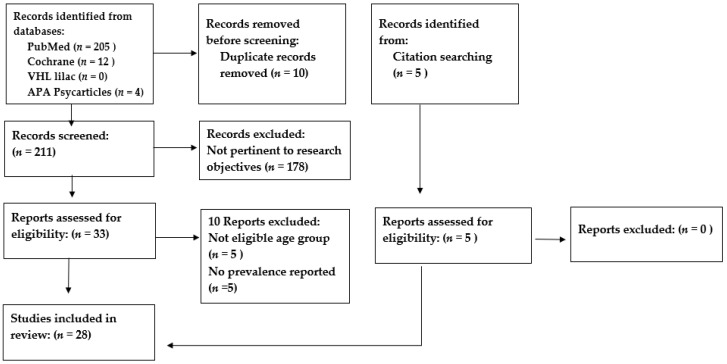
A flow chart detailing the number of studies included and excluded from the review.

Only studies that met the eligibility criteria were included in this review: (1) studies conducted among children and/or adolescents aged ≤19 years in the host and/or the refugee populations, (2) observational studies that reported prevalence data regarding mental health or psychosocial problems, and (3) studies conducted in Jordan. We excluded studies that were qualitative in design. Two authors independently screened the retrieved 211 titles and abstracts according to a pre-specified inclusion criterion. When the title or abstract did not clearly indicate whether an article should be included or excluded, the full article was read to determine if it met the inclusion criteria. Disagreement between the authors was solved by consensus, and the reasons for exclusion were recorded. A flow chart detailing the number of studies included and excluded at each stage is shown in Figure 1.

### 2.3. Data Extraction

Extraction was based on predefined data fields that included author and publication year, study design, study population, sample size, prevalence rate and associated factors, instrument(s) used, and main outcomes studied. One author extracted the data from the studies, and a second author checked the extracted data. Disagreement was resolved by a discussion between the two authors. When no agreement was reached, a third author was called upon to decide [13].

## 3. Results

### 3.1. Studies’ Characteristics

A total of 28 studies [7,8,9,10,14,15,16,17,18,19,20,21,22,23,24,25,26,27,28,29,30,31,32,33,34,35,36,37] were included in this review. Of those, 15 studies [14,15,16,17,18,19,20,21,22,25,29,33,35,36,37] examined mental health problems among Jordanian adolescents only, 3 studies were conducted among Syrian refugees [7,9,26], 3 studies included both Jordanian and Syrian adolescents [8,10,27], 2 studies were conducted among adolescents who resided in refugee camps [24,28], and 5 studies did not specify ethnicity [23,30,31,32,34]. The sample age ranged from 6–19 years. A total of 19 studies included data from schoolchildren [7,8,10,14,15,16,17,18,19,20,21,22,23,25,26,29,32,33,37], 2 studies included data from adolescents residing in refugee camps [24,28], 1 study included data from Syrian refugees living within the Jordanian community [9], 1 study included data from adolescents attending health clinics [34], 2 studies [35,36] included data from adolescents residing in institutional care centers, and 1 study included data from participants enrolled in a Mercy Corp registry [27]. Four studies examined female-only samples [15,18,20,24]. In total, 20 screening tools were used to assess mental health disorders and psychosocial problems (Table 1). Table 2 presents the summary of the studies’ findings.

### 3.2. Depression

A cross-sectional study of 1103 (605 females and 498 males) Jordanian adolescents aged 13–18 years attending public schools in Irbid reported that the prevalence of any mental disorder (mood disorders and anxiety disorders) was 28.6%, and the prevalence of mood disorders was reported to be 22.4% according to PHQ-A. Mood disorders included major depressive disorder (7.1%), dysthymic disorder (9.8%), and minor depressive disorder (11.2%) [17].

The prevalence of depressive symptoms was reported in 12 studies [9,10,16,17,19,21,23,24,31,34,36,37]. These studies used different tools to assess depression: the CES-C questionnaire [36], the CES-DC questionnaire [10,16], a modified version of the CES-C questionnaire used for children and adolescents, the CDI-2 questionnaire [9], the BDI-II [19], the PHQ-A [17], and a modified version of the PHQ-A, known as PHQ-9 [10]. The overall prevalence of depressive symptoms ranged from 7.1% to 73.8% [9,10,16,17,19,36] among adolescents. The prevalence ranged from 28.3% to 51.8% [9,10] among Syrian refugee adolescents and from 27.2% to 73.8% [10,16,17,19,36] among Jordanian adolescents. One study measured the prevalence of depressive symptoms among youths aged 7 to 18 years who reside in institutional care centers, which reported a rate of 45%. Five studies reported the severity of depression [19,21,23,24,31] using the BDI-II questionnaire [19,21,23,31] and the Depression, Anxiety, and Stress Scale (DASS) [24]. The prevalence of moderate depression ranged from 16.9%-19% [19,23,31], whereas severe depression ranged from 14.6–24% [19,23,31] and girls reported higher rates than boys [21]. The prevalence of moderate to extremely severe depressive symptoms was reported at 39.6% among married Palestinian girls aged 14–18 years who resided in refugee camps [24].

The CES-D and the CES-DC are both self-report depression questionnaires with 20 questions to rate depressive symptoms over the previous week. Each question had a score from 0 to 3 and the total score ranges from 0–60. Weissman et al. (1980) [38] the developers of the CES-DC, used a cut-off point of 15 as being suggestive of depressive symptoms in children and adolescents. Hence, scores over 15 suggest significant levels of depressive symptoms. Khader Y et al. [7] assessed the prevalence of depressive symptoms among Jordanian and Syrian adolescents using two screening tools: CES-DC and PHQ-9. According to both screening tools, Syrians reported higher rates of depressive symptoms compared to Jordanian adolescents, where findings from the CES-DC questionnaire showed a prevalence rate of 27.2% among Jordanians and 28.3% among Syrian refugees, and the findings from the PHQ-9 questionnaire revealed prevalence rates of 35.2% and 37.1% among Jordanians and Syrians, respectively. Malakeh M et al. [16] assessed the prevalence of depressive symptoms among Jordanian adolescents using the CES-DC questionnaire, which revealed a staggering prevalence of 73.8%. However, the cut-off point used by Malak was much lower compared to the cut-off point used by Khader Y et al. [10] (≥15 vs. >30). Alassaf A et al. [34] also leveraged the CES-DC questionnaire to assess the prevalence of depressive symptoms among adolescents aged 10–17 years, with type 1 diabetes (T1D). Adolescents who attended the Pediatric endocrine clinic at Jordan University Hospital from February 2019 and February 2020 were asked to fill out the self-reported questionnaire. Out of the 108 children enrolled, 50 children (46.3%) had a depression score of 15 or more.

The different studies assessed the factors associated with depression. Among Jordanian and Syrian refugee adolescents, female gender [10,16,17,19,23,31,34,37], older age [19,23,37], residing in families with a monthly income of less than JD 300 and a residing in families with low monthly incomes, reporting a chronic health problem, a mental health problem, a learning difficulty, or a psychiatric diagnosis [19,37], not having resilient traits [9,10], and having high emotional and peer relation problems were associated with a higher risk of developing depressive symptoms [10]. Among the Jordanian adolescents, older students [21], those with a parental history of mental health disorders [17], poor or medium academic achievement (<70%) [16], a family income of less than JD 300 (USD 423), reporting a chronic health problem [19], a mental health problem [19], a learning difficulty [19,21], a psychiatric diagnosis, and seeking psychological help in the past were at a higher risk of developing depressive symptoms [19]. Among the Syrian refugee adolescents experiencing more traumatic life events was associated with higher depression scores [9]. Internet addiction [16], previous trauma, and father’s educational level [24] were predictors of depression [16] and experiencing difficulties with online education during COVID-19 were reported as predictors of both depression and anxiety [23].

### 3.3. Anxiety

Four studies [10,16,17,24] reported the prevalence rates of anxiety among adolescents. One study reported a prevalence of 41.5% and 46.8% among Jordanian and Syrian adolescents, respectively, using the GAD-7 screening tool [10]. The second study reported a prevalence of 16.3% among Jordanian adolescents using the PHQ-A screening tool, and the third reported a prevalence of 42.1% among Jordanian adolescents using the CES-DC tool. The PHQ-A questionnaire, which was used by Alslman E et al. [17], was not based on total scores, but instead on specific conditions or responses which should be met to provide a provisional diagnosis. According to Khader Y et al.’s study, Syrian refugee adolescents compared to Jordanian adolescents suffered from higher prevalence rates of anxiety (41.5% vs. 46.8%). The fourth study assessed the severity of anxiety and stress symptoms among Palestinian married girls who resided in refugee camps. Using the DASS tool, it was noted that 35.6% and 9.8% suffered from moderate to extremely severe anxiety and stress symptoms, respectively [24].

Jordanian and Syrian refugee adolescents who reported high emotional symptoms, high conduct problems, and hyperactivity disorders were more likely to develop anxiety [10]. Factors associated with higher rates of anxiety among Jordanian adolescents included female gender, older students [16,17], having a family income of <JD 250 (USD 352) [16], a parental history of mental disorders [17], medium or poor academic achievement (<70%), and having a severe level of Internet addiction [16]. Female adolescents experienced higher severity levels of anxiety compared to males [21,23]. Feeling safe was reported as a protective factor among Jordanian and Syrian refugee adolescents, and high perceived support among Jordanians was reported as a protective factor [10].

Attention deficit hyperactivity disorder was examined in two studies [22,29], and the authors assessed the prevalence of ADHD and its subtypes among Jordanian adolescents aged 6–12 years. The prevalence of ADHD as reported by teachers ranged from 19.2–40.6% [22,29], and 13.0% as reported by parents [29]. Girls and older children experienced lower rates of ADHD and its subtypes. Respectively, the prevalence rate of ADHD subtypes among 6–9-year-old and 10–12-year-old children were 10.8% and 6.3% for inattention, 15.2% and 5.2% for hyperactivity–impulsivity, and 32.4% and 10.7% for the combined subtype [22].

### 3.4. Post-Traumatic Stress Disorder, Emotional and Behavioral Problems

Four studies reported the prevalence of PTSD among adolescents [7,26,27,36]. One study assessed the prevalence of PTSD among Syrian refugees and Jordanian non-refugee adolescents that were enrolled in a Mercy Corps registry of youth [27]. The study utilized the Child Revised Impact of Events Scale to measure its prevalence and it was reported at 65.1% and 16.2% among Syrian refugees and Jordanian non-refugees, respectively. Two studies assessed the prevalence of PTSD severity among Syrian refugee adolescents [7,26], utilizing the Child Post Traumatic Stress Disorder Symptom Scale (CPSS) (cut-off point of ≥21) [7] and the PTSD Checklist—Civilian Version (PCL-C) [26]. Beni Y et al. reported the prevalence rate of moderate to severe symptoms at 31% [7], while Ramadan M et al. reported the prevalence of moderate and severe symptoms at 46.9% and 29.7%, respectively [26]. Across the above-mentioned studies, the prevalence was higher among females and among adolescents of whom at least one of their parents had died. Prevalence did not differ according to total family income, family size [7] or spirituality levels [26]. One study [36] reported a PTSD prevalence rate of 24% among youths aged 7–18 years, who resided in five institutional care centers in Jordan. The study used the University of California, Los Angeles, PTSD Index for DSM-IV (UPID) to report the PTSD prevalence rate. Close peer relationships and reports of abuse were statistically significant predictors of PTSD [36]. Youths who reported abuse were 5.9 times more likely to suffer from PTSD, compared to youths who did not report abuse [36]. Youths who reported having close peer relationships had 93% lower odds of PTSD, compared to youths who did not report having close peer relationships [36].

Three studies assessed the emotional and behavioral problems among adolescents [8,25,35], where two questionnaires were used: the strengths and difficulties questionnaire (SDQ) [8,25] and the CBCL [35]. In two of the studies where the SDQ was used, a score of 20–40 was indicative of abnormal levels of total difficulties (including emotional, conduct, hyperactivity/inattention, and peer problems as well as prosocial behavior) [8,25], whereas a score of more than 65 on the CBCL questionnaire indicated a borderline clinical cut-off and a score of more than 69 indicated a clinical cut-off [35]. The prevalence of overall total difficulties among Jordanian adolescents ranged significantly from 11.7% to a substantial 52.5% [8,25]. The Syrian adolescents had slightly higher levels of total difficulties compared to the Jordanian adolescents (58.2% vs. 52.5%) [8]. Lower academic achievement was associated with greater emotional and behavioral problems [25] and girls had a higher risk of developing emotional symptoms and hyperactivity/inattention problems than boys had [8,25]. In contrast, the males had higher scores on the peer problems subscale [25]. The Syrian adolescents were more likely to develop overall difficulties and emotional symptoms but had lower rates of peer relationship problems [8]. Prosocial behavior was negatively correlated with emotional symptoms (r = −0.332, *p* < 0.001), conduct problems (r = −0.415, *p* < 0.001), and peer relationship problems (r = −0.239, *p* = 0.001) [8].

### 3.5. Eating Disorders

A total of four studies [14,15,18,33] assessed the prevalence of eating disorders among Jordanian adolescents. Two studies [15,18] were conducted among females only, and two studies [14,33] involved female and male adolescents. Al kloub I et al. [14], Alfoukha M et al. [18], and Al-Sheyab N et al., [33] utilized the EAT-26 questionnaire (cut-off point ≥ 20) to assess the prevalence of eating disorders and reported a prevalence of 40.4%, 12%, and 23.6% among adolescents, respectively. Mousa Y et al. [15] used the Eating Habits Questionnaire and reported a prevalence of 33.4%. Only one study reported the prevalence of specific types of eating disorders which included bulimia nervosa (0.6%) and binge eating disorders (1.8%), and no anorexic cases were found [15]. Two studies [14,20] assessed the prevalence of body shape dissatisfaction among Jordanian adolescents which utilized the BSQ screening tool. Al Kloub I et al. [14] reported a prevalence of 16.8% among female and male adolescents, and Mousa Y. et al. [20] reported a prevalence at 21.2% among female adolescents.

Female gender [14,33], place of residence (urban more often than rural) [14], excess weight [14,15], distorted perception of weight [14], body shape dissatisfaction [14,15,18], peers’ influence [14,15,18,33], parents’ influence, mass media influence [14,18], increased psychological distress, low self-esteem [18], and high family socio-economic status [14] were associated with the increased prevalence of disordered eating. Body shape dissatisfaction, low self-esteem, negative peer pressure, and being young were significant predictors of the risk of eating disorders [18]. Body shape dissatisfaction was associated with excess weight [14,20], a distorted perception of weight [14], negative eating attitudes, media influence, and peer pressure [20].

## 4. Discussion

This review highlights the magnitude of mental health disorders and psychosocial problems among children and adolescents living in Jordan. Among the 28 studies included in this review, the prevalence rates among adolescents were 7.1% to 73.8% for depression [9,10,16,17,19,36], 16.3% to 46.8% for anxiety [10,16,17], 13.0–40.6% for ADHD [22,29], 11.7–55.2% for overall emotional and behavioral difficulties [8,25,35], 16.2–65.1% for PTSD [27], and 12–40.4% [14,15,18] for eating disorders. Although there is wide variation among the reported prevalence rates, it is evident that the rates are high. However, the generalizability of the findings is limited because the studies did not include children of all ages and from all regions of the country, as well as the variety of screening tools that were used, in addition to the different study methodologies. Despite a sufficient number of studies published between 2010–May 2023, several publications [7,8,10,15,19,20,21,37] were derived from the same parent study and dataset, hence sharing the same methodology, but reporting different outcomes and/or variables.

Although the prevalence rate of anxiety among Jordanian adolescents was high, Syrian refugee adolescents suffered even higher rates compared to Jordanians. This can be attributable to the numerous traumatic and stressful events that refugees have been subjected to since the Syrian civil war (2011), such as displacement, separation from family, and death of a family member [39,40]. Adolescents’ mental health and psychosocial well-being is also affected by post-conflict conditions in host countries, such as community acceptance, school attendance, family support [41] and differences in cultural, political, and social contexts [42]. Exposure to such harsh conditions increases the likelihood of developing mental health problems [43], which was evident in the high rate of PTSD among the Syrian refugees (65.1%). Alternatively, a lower rate of PTSD (35.1%) was reported among the Syrian adolescents, who were internally displaced in Syria because of the war [44].

It was seen that the prevalence rate of mental health issues among adolescents was higher in cities that were affected by the influx of refugees compared to the capital city, Amman. For example, the prevalence of emotional and behavioral problems among Jordanian adolescents attending schools in cities that are densely populated by refugees was much higher [8] compared to students attending schools in Amman [25] (55.2% vs. 11.7%), in addition to the variation in the prevalence rate of ADHD among students attending schools in Mafraq (40.52%) [22] vs. Amman (19.2%) [29]. However, these findings should be interpreted with caution, due to the different tools used and different sampling techniques. ADHD is one of the most common and well-studied psychiatric disorders in the pediatric population [45]; however, there still remains a limited base of evidence regarding the prevalence rate and its associated factors in Jordan, in addition to the high variability in the prevalence rates that were reported.

The two studies [10,16] that used the CES-DC tool to assess the prevalence rate of depressive symptoms among adolescents, used different cut-off points (≥15 vs. >30). Therefore, the higher prevalence rate of depressive symptoms among Jordanians compared to Syrian refugees is attributable to the difference in the cut-off points used. In addition, the difference in the cut-off points used can explain the wide range of prevalence rates among Jordanians (27.2–73.8%).

Comparisons with other studies in the Middle East and North Africa (MENA) region should be made with caution since the studies used different screening tools to assess mental health issues or different sampling procedures or investigated different populations. However, a high prevalence of depressive symptoms was reported in two studies conducted in Egypt [46] and Sudan [47]. One study used the CDI tool (cutoff of ≥ 24) and reported a high prevalence of depressive symptoms among adolescent females, aged 14–17 years, residing in Egypt (15.3%) [46]. Another study used the BDI tool (cut-off point ≥ 8) and reported a prevalence rate of 28.2% among adolescent females, aged 12–19 years, residing in Sudan [47].

In our review, we found that multiple factors were associated with depression and anxiety among adolescents, such as having a parent with a mental health condition, older age, and female gender. These findings are consistent with reviews from low- and middle-income countries [41,48,49]. In addition, females were more likely to develop anxiety [16,17], depression [10,16,17,19], PTSD [8], and eating disorders [14] compared to male adolescents. A systematic review examining the prevalence rate of depressive symptoms among adolescents globally, reported a higher prevalence rate among female adolescents than male adolescents (34% vs. 24%) [50]. Another factor that influenced the prevalence rate of mental health disorders was income level [51]. There was some contradiction in the findings, where two studies showed that a low-income level was associated with the increased prevalence of mental health disorders [14,19], such as depression and anxiety, while another study showed that a high family socioeconomic status increased the prevalence of eating disorders [16]. Further studies with this focus are needed to deepen the knowledge about the subject.

The high overall prevalence rates reported in this review should be viewed with caution because female adolescents were overrepresented in the total population of the included studies. Females are more likely to be exposed to risk factors such as sexual abuse [52], gender discrimination [53], and violence [54], as well as experience chronic concern with their appearance and body dissatisfaction [55], which makes them more vulnerable to developing and internalizing mental health problems [56]. Future research should clarify how gender differences affect the trajectory of mental health among children and adolescents and provide empirical evidence to healthcare specialists and policymakers to translate the knowledge of gender differences into culturally sensitive program implementation. Further, all of the included studies utilized self-reported screening tools, which are subjected to self-recall bias. Screening tools, compared to diagnostic interviews, have been shown to overestimate the prevalence rates of mental health disorders [41].

This review indicates that there has been an increase in the number of epidemiological studies examining mental health problems among adolescents in Jordan over the years. However, there remains a scarcity of studies investigating the prevalence of mental health disorders in Jordan, specifically among refugees. None of the studies assessed the prevalence of eating disorders among refugees, as well as sleeping disorders and substance use disorders among adolescents. Moreover, mental disorders’ etiologies, including the COVID-19 pandemic’s impact, and their consequences were not thoroughly examined, as well as the help-seeking behaviors among children and adolescents regarding mental health services. Additional research regarding health-related quality of life (HRQOL), and drug addiction among adolescents experiencing mental health problems should also be investigated to assess the impact of mental health disorders.

Our study is limited by the fact that only four databases were searched; therefore, studies indexed in other databases were missed during the search strategy. In addition, our search strategy might not have extracted all pertinent studies from the four databases; therefore, we might have missed some relevant studies. Moreover, the included studies all had different methodologies and target audiences and used a different sampling approach and self-reported tools to measure the prevalence of mental health issues. Therefore, our results and conclusions should be interpreted with caution.

## 5. Conclusions

In conclusion, this review has highlighted the magnitude of mental health disorders and psychosocial problems among children and adolescents. The findings should be interpreted with caution due to the heterogeneity of the studies, which was outlined in the variety of screening tools and cut-off points that were used, as well as the lack of representative samples. Several factors were associated with mental health issues. Syrians compared to Jordanian adolescents were more likely to develop anxiety symptoms. Female gender and older age were associated with higher rates of mental health problems. Further studies are needed to investigate other factors such as drug addiction, quality of life, and help-seeking behaviors among adolescents experiencing mental health issues, as well as etiologies and trajectories of mental health disorders. Future research should use a robust methodological framework to come up with thorough evidence to inform policies.

## Figures and Tables

**Table 1:** Mental health screening tools used in the retained studies.

Name of the Tool	Main Outcome(s) Studied
The Child Post Traumatic Stress Disorder Symptom Scale (CPSS)	Post traumatic stress disorder
PTSD checklist—Civilian Version (PCL-C)	Post traumatic stress disorder
The Child Revised Impact of Events Scale	Post traumatic stress disorder
The University of California, Los Angeles, PTSD Index for DSM-IV (UPID)	Post traumatic stress disorder and exposure to traumatic events
Center for Epidemiologic Studies—Depression Scale (CES-D)	Depression (levels of depressive symptoms)
The Center for Epidemiologic Studies—Depression Scale for Children (CES-DC)	Depression (levels of depressive symptoms)
Patient Health Questionnaire for Adolescents (PHQ-A)	Mood disorders (major depressive, dysthymic, and minor depressive disorders) Anxiety disorders (panic and generalized anxiety disorders)
Patient Health Questionnaire-9 (PHQ-9) (Modified version of PHQ-A)	Depression (significant levels of depressive symptoms)
Generalized Anxiety Disorder Questionnaire (GAD)	Anxiety
The Strength and Difficulties Questionnaire	Behavioral and emotional symptoms (emotional problems conduct problems, hyperactivity/inattention, peer problems, and prosocial behavior).
Symptom Checklist Anxiety	Anxiety
Eating Attitude Test (EAT-26)	Eating disorders
Body Shape Questionnaire (BSQ)	Body shape dissatisfaction
Eating Habits Questionnaire	Eating disorders
Beck’s Depression Inventory-II (BDI-II)	Depression
Children’s Depression Inventory 2 (CDI)	Depression
Depression, Anxiety, and Stress Scale	Depression, anxiety and stress
Attention Deficit Disorder Evaluation Scale (ADDES)—school version and parental questionnaire	Attention deficit hyperactivity disorder (inattentive, hyperactive–impulsive, and combined subtypes)
Arabic version of the DSM-IV rating scale for the diagnosis and classification of attention deficit hyperactivity disorder	Attention deficit hyperactivity disorder (inattentive, hyperactive–impulsive, and combined subtypes)
Child Behavioral Checklist (CBCL)	Behavioral problems (externalizing and internalizing problems) Suicidality was measured by either of the following two items in the CBCL: Item 91: “I think about killing myself” or self-harm behavior. Item 18: “I deliberately try to hurt or kill myself”

**Table 2:** Summary of the results.

Author, Publication Year	Study Design	Study Population	Sample Size	Prevalence Rate	Associated Factors
Yonis, B. et al. 2020 [7]	Cross-sectional	Syrian schoolchildren aged 12–18 years enrolled in schools of four cities: Mafraq, Sahab, Ramtha, and Zarqa. Mean age 14.5 (SD = 1.5) years.	1768 (991 females and 777 males)	The prevalence of moderate to severe PTSD * (cut-off point ≥ 21) was 31% (34.3% in females and 26.8% in males)	Having lost at least one parent (OR = 1.7 (95% CI 1.2–2.5)) and female gender (OR = 1.5 (95% CI 1.2–1.9)) were associated with increased odds of moderate to severe PTSD
Yonis, B. et al. 2021 [8]	Cross-sectional	Jordanian schoolchildren and Syrian refugee schoolchildren, aged 12–17 years, enrolled at schools in Mafraq, Ramtha, and Zarqa. Mean age 14.7 (SD = 1.6) years.	3645 schoolchildren (1877 Jordanian and 1768 Syrian schoolchildren refugees (females 2013; males 1632)	Abnormally high total difficulty score of 55.2% (58.2% among refugees and 52.5% among Jordanians), peer relationship problems (53.6% of Jordanians and 55.5% of Syrians), conduct problems (47.6% among refugees and 44.8% among Jordanians), emotional symptoms (32.0% among refugees and 30.8% among Jordanians), hyperactivity/inattention problems (35.5% among refugees and 36.9% among Jordanians), and prosocial behavior problems (42.5% among refugees and 43.0% among Jordanians)	Syrian adolescents were more likely to develop overall difficulty problems (OR: 1.431, 95% CI: 1.071–1.912) and emotional symptoms (OR: 1.156, 95% CI: 1.007–1.326) and were less likely to develop peer relationship problems (OR: 0.791, 95% CI: 0.631–0.992). Those who had a history of parental death scored significantly higher on the overall difficulties, emotional symptoms, and conduct problems scales. School children who experienced parental separation had significantly more peer relationship problems (71.5%) and more prosocial behavior problems (54.4%) than those with parental death (60.2% and 40.4%, respectively) and who lived with both parents (52% and 41.5%, respectively).
Dehnel, Rebecca. et al. 2022 [9]	Cross-sectional	Syrian refugee children aged 10–17 living within the Jordanian community in Irbid and Ramtha. Mean age 13.4 years	339 (85 males, 252 females, and 2 unreported)	A slight majority of children (51.8%) met the criteria for having significant depressive symptoms (cut-off score of 13), and 40.4% met the cut-off score of 16. All children (100%) have been exposed to at least one traumatic event (car accidents, robberies, and war experience). In total, 27.8% of children experienced suicidal ideation	There was a significant negative correlation between resilience and depression scores (−0.33, *p* < 0.001) and a positive but weak correlation between traumatic life events and depression (CDI-2) (r = 0.1, *p* = 0.02)
Khader, Y. et al. 2021 [10]	Cross-sectional	Jordanian and Syrian refugee adolescents aged 12–17 years, who were registered at schools in four cities, Mafraq, Sahab, Ramtha, and Zarqa. Mean age 14.8 (SD = 1.8) for the Jordanian adolescents and 14.5 (SD = 1.5) for the Syrian adolescents	1877 (1021 females and 856 males) Jordanian adolescents and 1768 (991 females and 777 males) Syrian refugee adolescents	The prevalence of depressive symptoms using CES-DC* among Jordanian and Syrian adolescents was 27.2% and 28.3%, respectively. The prevalence of depressive symptoms using PHQ-9* among Jordanian and Syrian adolescents was 35.2% and 37.1%, respectively. The prevalence of generalized anxiety disorder using GAD-7* among Jordanian and Syrian adolescents was 41.5% and 46.8%, respectively.	Jordanian female adolescents (OR:1.6, 85% CI: 1.2–2.1), Syrian female adolescents (OR:1.4, 85% CI: 1.4–2.5), not feeling safe among Jordanians (OR:2.2, 85% CI: 1.5–3.4) and Syrians (OR:1.8, 85% CI: 1.2–2.8), not having resilient traits among Jordanians (OR:1.9, 85% CI:1.4–2.6) and Syrians (OR:1.8, 85% CI:1.3–2.4), having high emotional symptoms among Jordanians (OR:1.9, 85% CI:1.4–2.6) and Syrians (OR:1.9, 85% CI: 1.4–2.6), and high peer relation problems among Jordanians (OR:1.6, 85% CI: 1.2–2.2) and Syrians (OR:1.4, 85% CI: 1.0–1.8) were associated with higher odds of depressive symptoms
Al-Kloub, I. et al. 2019 [14]	Cross-sectional	Jordanian school children aged 15–18 years attending public secondary schools in Amman, Al-Zarqa, and Al-Karak. Mean age 16.01 (SD = 0.72) years	963 (460 males and 503 females)	Disordered eating (40.4%) and body shape dissatisfaction (16.8%)	Female gender (OR:1.30 95% CI: 1.0–1.92), urban residence (OR:3.70 (2.68–4.47), excess weight (OR:1.86 (1.2–3.88), distorted perception of weight (OR:5.08 (1.39–11.80), body shape dissatisfaction (OR:4.67 (3.10–7.03), parents’ influence (OR:1.29 (1.10–2.60), peers’ influence (OR:1.55 (1.34–2.18), and mass media influence (OR:2.30 (1.42–3.93) were associated with disordered eating
Mousa, Y. et al. 2010 [15]	Cross-sectional	Adolescents’ schoolgirls aged 10–16 years attending elementary public and private schools in Amman. Mean age 12.9 (SD = 1.8) years	326 females	Eating disorders (33.4%) bulimia nervosa (0.6%), binge eating disorders (1.8%), and eating disorders not otherwise specified (31%)	Participants who had dwelling relatives (RR: 2.2 (0.99–4.8)), and friends with an ED history (RR: 3.9 (1.0–15.6)) were at a significantly higher risk of having an ED. The likelihood of developing an ED significantly increased among participants who had body image dissatisfaction (RR: 5.2 (3.3–8.4)), who were post-menarcheal (RR: 1.6 (1.4–1.9)), and overweight or at risk of being overweight (RR: 1.91 (1.4–2.6))
Malak, M. et al. 2018 [16]	Cross-sectional	Jordanian school children aged 12–18 years attended public schools in Amman. Mean age 14.92 (SD = 1.69) years	800 (400 females and 400 males).	Anxiety (42.1%) and depression (73.8%)	Age 16 to younger than 17 years, female gender, and a family income of <JD 250 were associated with a higher prevalence of anxiety. Medium or poor academic achievement and having a severe level of Internet addiction were associated with anxiety and depression
Alslman, E. et al. 2017 [17]	Cross-sectional	Adolescent students aged 13–18 years attending public schools in Irbid. Mean age 15.27 (SD = 0.937) years.	1103 (605 females; 498 males)	Any mental disorder (28.6%), mood disorders (22.4%) which included major depressive disorder (7.1%), dysthymic disorder (9.8%), and minor depressive disorder (11.2%), anxiety disorders (16.3%) which included generalized anxiety disorders (12.4%) and panic attack disorder (9.4%)	Females (OR = 2.4; 95% CI: 1.77–3.25), older students (OR = 1.34; 95% CI: 1.15–1.57), and students whose parents (one or both) had mental disorders (OR = 4.67; 95% CI: 2.85–7.65) were more likely to suffer from mental disorders. Students who were living with one parent or people other than their parents were less likely to have mental disorders than those who were living with both parents (OR = 0.15; 95% CI: 0.03–0.85)
Alfoukha, M. 2019 [18]	Cross-sectional	High schoolgirls aged 16–18 years from governmental and private schools in the central region of Jordan. Mean age 16.9 (SD = 0.61) years.	799 females	The prevalence of the risk of eating disorders was 12%. Approximately 34.5% had low or high self-esteem, and 65.5% reported moderate self-esteem	The risk of eating disorders had a significant and positive correlation with body shape dissatisfaction (r = 0.41), self-esteem (r = 0.19), psychological distress (r = 0.15), and pressure from family (r = 0.27), peers (r = 0.26), and the media (0.23).
Dardas, A. et al. 2018 [19]	cross-sectional,	Nationally representative school sample of Jordanian adolescents aged 12–17 attending private or public school. Mean age 15.0 years (SD = 1.5)	2349 (1389 females; 960 males)	Approximately 47% with minimal depression, 18% with mild depression, 19% with moderate depression, and 15% with severe depression. The prevalence of moderate to severe depression scores was 34%	The mean severity of depression total scores was significantly higher in the following groups of adolescents: females; those aged 14 and older; those residing in families with monthly incomes less than JD 300 (USD 423); those reporting a chronic health problem; those reporting a mental health problem; those reporting a learning difficulty; those reporting a psychiatric diagnosis; and those reporting seeking psychological help in the past
Dardas, A et al. 2018 [37]					
Mousa, Y. et al. 2010 [20]	Cross-sectional	Adolescents schoolgirls, aged 10–16 years, attending public and private schools in Amman. Mean age of 12.9 (SD = 1.8) years	326 females	Approximately 21.2% of participants displayed body image dissatisfaction (BID), and 40.5% of adolescent girls had negative eating attitudes	Participants who were overweight or at risk of being overweight (RR: 2.8 (2.1–3.8)_ and engaged in negative eating attitudes (RR: 3.6 (2.9–4.5)) were at a significantly higher risk of developing BID. Living in a Western country is inversely associated with BID (RR: 0.34 (0.12–1.1)). Media influence, such as exercise or going on a diet to lose weight because of a magazine article or picture (OR:1.6, 95% CI: 1.3–1.9) and making efforts to look like females in the media (1.2, 1.1–1.4), were associated with BID. Pressure from parents (1.9, 1.6–2.3) and peers (peer pressure) (1.6, 1.3–1.98) were associated with the increased rate of BID
Dardas, A. et al. 2021 [21]	Cross-sectional	Jordanian adolescents aged 12 to 17 using a nationally representative school survey	2349 (1389 females; 960 males)	Approximately 41% of girls and 26% of boys reported scores indicating moderate to severe depression	Among female adolescents, the severity of depression total scores was significantly related to the following characteristics: age, family monthly income, mental health problems, and learning difficulties. Male adolescents’ depression scores were significantly related to their age, mental health problems, and learning difficulties
Al Azzam, M. et al. 2017 [22]	Cross-sectional	Jordanian adolescents aged 6–12 years enrolled in public primary schools in Mafraq	480 (250 males; 230 females)	The prevalence of ADHD* was 40.62%. The prevalence rates within the inattentive, hyperactive–impulsive, and combined subtypes were 10.83, 9.58, and 20.21%, respectively.	Older children, boys, and increased family size were associated with the increased prevalence of ADHD symptoms.
AlAzzam, M. et al. 2021 [23]	Cross-sectional	Senior high school students aged 17–18 years old	384 (153 males; 230 females)	The prevalence of mild to severe depression was 72.4%, while the prevalence of moderately severe depression was 16.9% and 14.6% for severe depression. The prevalence of mild to severe levels of anxiety was 74.9%, while 16.7% reported having severe anxiety and 58.1% reported mild to moderate levels of anxiety	Females and non-working students were associated with higher scores for depression. Females and non-working students reported higher anxiety mean scores
Malakeh M. et al. 2021 [24]	Cross-sectional	Teenage Palestinian married girls residing in Palestinian refugee camps, aged 14–18 years. Mean age 16.9, (SD = 0.96) years	205 females	Approximately 39.6%, 35.6%, and 9.8% suffered moderate to extremely severe levels of depression, anxiety, and stress symptoms, respectively.	Age, exposure to previous trauma and parents’ educational level were associated with all mental health symptoms
Atoum M. et al. 2018 [25]	Cross-sectional	Jordanian adolescents attending two public secondary schools located in Amman, aged 14–16 years. Mean age 15.12 years (SD = 0.80).	810 (374 males; 436 females).	The overall prevalence of emotional and behavioral problems was 11.7%. The prevalence of emotional symptoms, conduct problems, hyperactivity, prosocial behavior, and peer problems was 14.2%, 12.5%, 7.5%, 4.2%, and 5.7%, respectively	Lower academic achievement was correlated with greater emotional and behavioral problems
Ramadan M. et al. 2022 [26]	Cross-sectional	Syrian adolescents who attended Jordanian schools in the northern region, aged 12–17 years. Mean age 14.89 (SD = 1.34)	418	The prevalence rates of moderate and severe levels of PTSD were 46.9% and 29.7%, respectively	Spirituality levels were not associated with PTSD severity levels
Chen, A. et al. 2019 [27]	Cross-sectional	Syrian refugee and Jordanian non-refugee adolescents enrolled through a Mercy Corp registry, aged 12–18 years. Mean age 14.29 (SD = 1.79)	450 (240 Syrian refugees; 210 Jordanian non-refugees) (189 females; 261 males)	The prevalence of PTSD was 65.1% among the Syrian refugee adolescents and 16.2% among the Jordanian non-refugee adolescents	-
Itani, T. et al. 2017 [28]	Cross-sectional	Palestinian students aged 13–15 years residing in the Occupied Palestinian Territory and United Nations Relief Works Agency camps (UNRWA)	Total *n* = 14303, 1495 from Jordan	Suicidal ideation was assessed by one item: “During the past 12 months did you ever seriously consider attempting suicide?” Suicidal planning was assessed by one item: “During the past 12 months, did you make a plan about how you would attempt suicide?” In the Jordanian UNRWA camps, the prevalence of suicidal ideation and suicidal planning was 21.7% and 18.1%, with a pooled global prevalence of 19.9% and 17.1%, respectively. The prevalence of suicidal thinking (ideation/planning) was 27.0% with a pooled global rate of 25.6%	A history of marijuana use (OR 5.1 95% CI 2.9–10.6), no close friends (OR 2.9 95% CI 2.0–4.2), recent tobacco use (OR 3.2 95% CI 2.5–4.1), food insecurity (OR 2.4 95% CI 1.7–3.6), and being bullied recently (OR 2.1 95% CI 1.6–2.6) were associated with higher odds of having suicidal ideation/planning in Jordan
Nafi, O. et al. 2020 [29]	Cross-sectional	Adolescents attending a total of three schools in Amman and Karak, aged 6–12 years	1326 students (712 males; 614 females)	The prevalence of ADHD and its subtypes as reported by teachers and parents are the following: ADHD (19.2% vs. 13.0%), attention deficit (7.3% vs. 4.5%), hyperactive–impulsive (7.8% vs. 6.4%), and combined (4.2% vs. 2.1%), respectively	-
Al Rahamneh, H. 2021 [30]	Cross-sectional (online survey)	Parents with children between the ages of 5 and 11 years old	1309 parents (593 with female children; 716 with male children)	According to parents’ reports, children were more irritable (66%), more likely to argue with the rest of their family (60.7%), be nervous (54.8%), reluctant (54.2%), lonely (52.4%), angry (51.8%), restless (48.6%), cry easily (47.3%), have difficulty concentrating (46.1%), be anxious (44.8%), dependent on parents (44.2%), sad (43.4%), uneasy (42.9%), frustrated (42.7%), worried (38.7%), and were afraid of COVID-19 infection (38.6%) during the lockdown compared to the pre-COVID-19 period	The child’s age, parental marital status, and fathers’ employment status were associated with emotional and behavioral differences before and during the COVID-19 pandemic
Dardas, L. et al. 2018 [31]	Cross-sectional	Adolescents aged 12–17 years (pilot study)	88 (35 females; 53 males)	The prevalence of mild, moderate, and severe depression were 22%, 19%, and 24%, respectively.	Females experienced significantly higher levels of depression than males
Hamdan-Mansour, A. et al. 2020 [32]	Cross-sectional	Adolescents enrolled in public high schools in the central district of Jordan, aged 13–19 years	1497 (753 females; 744 males)	Approximately 8.5% used stimulants, 12.8% used tranquilizer sedatives, 14.2% used hypnotic agents, 5.5% used antidepressant agents, 18.3% smoked cigarettes, and 31.4% smoked water pipes	High psychological stress, low coping efficacy, and low perceived social support from family were associated with a higher risk of substance use
Al-Sheyab Nihaya, A. et al. 2018 [33]	Cross-sectional	Jordanian school children attending private or public schools in Northern Jordan, aged 14 years to 16 years old, from both genders from grades 8 to 10. Mean age (SD) 15.06 (0.8) years	738 (408 females; 330 males)	The prevalence of disordered eating behavior (DEB) was 23.6% (29.4% among females and 16.4% among males, *p* < 0.000)	-
Alassaf, Abeer et al. 2023 [34]	Cross-sectional	Adolescents aged 10–17 years with type 1 diabetes. Mean age of 13.7 ± 2.3 years	108 (49 males; 59 females)	Approximately 50 (46.3%) adolescents had a depression score of 15 or more on the CES-DC, indicating significant depressive symptoms	Girls were more likely to have a depression score of 15 or more compared to boys (OR = 3.41, *p* = 0.025). Patients who rarely self-monitored their blood glucose levels were more likely to have a depression score of 15 or more compared to those who tested regularly (OR = 36.57, *p* = 0.002)
Gearing, Robin E. et al. 2013 [35]	Cross-sectional	Jordanian adolescents residing in four institutional care centers. Mean age was 12.26 (SD = 2.2 years)	70 (39 males; 31 females)	Approximately 53% experienced mental health problems, and 43% and 46% had high internalizing and externalizing scores, respectively	Male gender, care entry because of maltreatment, time in care, and transfers were the most significant predictors of problems
Gearing, Robin E et al. 2015 [36]	Cross sectional	Jordanian children aged 7–18 years residing in five institutional care centers	86 (41 Females; 45 males)	45% had depression, 24% had PTSD, and 27% had suicidality	Youths who reported abuse were 5.9 times more likely to suffer from PTSD compared to youths who did not report abuse. Youths who reported having close peer relationships had 93% and 88% lower odds of PTSD and depression, respectively, compared to youths who did not report having close peer relationships

* OR = odds ratio, ADHD = attention deficit hyperactivity disorder, PTSD = posttraumatic stress disorder, CES-DC = Center for Epidemiological Studies Depression Scale for Children, GAD-7 = Generalized Anxiety Disorder 7, PHQ-9 = Patient Health Questionnaire 9, and CI = confidence interval.

## Data Availability

Not applicable.
